# Attenuated antigen-specific T cell responses in cirrhosis are accompanied by elevated serum interleukin-10 levels and down-regulation of HLA-DR on monocytes

**DOI:** 10.1186/1471-230X-13-37

**Published:** 2013-02-27

**Authors:** Jack Peter, Oliver Frey, Andreas Stallmach, Tony Bruns

**Affiliations:** 1 Department of Internal Medicine IV, Jena University Hospital, Friedrich Schiller University Jena, Jena, Germany; 2 Institute of Clinical Chemistry and Laboratory Diagnostics, Jena University Hospital, Jena, Germany; 3 Integrated Research and Treatment Centre - Centre for Sepsis Control and Care (CSCC), Jena University Hospital, Friedrich Schiller University Jena, Jena, Germany; 4 NIHR Biomedical Research Unit and Centre for Liver Research, Institute of Biomedical Research, University of Birmingham, Edgbaston, Birmingham, B15 2TT, United Kingdom

**Keywords:** Cirrhosis, Adaptive immunity, Cellular immunity, Virus-specific T cell responses, Bacterial translocation, Interleukin-10

## Abstract

**Background:**

Advanced liver disease predisposes to bacterial translocation and endotoxaemia which can contribute to elevated circulating levels of IL-10 and down-regulation of MHC class II on antigen-presenting cells. We sought to evaluate antigen-specific T-cell responses toward common viral antigens in order to investigate defects in cellular immunity in cirrhosis.

**Methods:**

Peripheral blood was obtained from 22 cirrhotic patients with systemic inflammation, 13 cirrhotic patients without systemic inflammation and 14 healthy controls. C-reactive protein was used as an indicator for systemic inflammation using a cut-off of 10 mg/l. Intracellular Th1 cytokines were quantified after T cell-stimulation with the viral peptides EBNA1 and BZLF1 or the bacterial superantigen SEB by flow cytometry. Serum levels of lipopolysaccharide-binding protein (LBP) and IL-10 were quantified by ELISA.

**Results:**

Compared to healthy controls, patients with cirrhosis had higher circulating levels of LBP and IL-10, an expansion of peripheral blood CD14^+^ monocytes with low HLA-DR expression and an increased fraction of CD25-positive CD4^+^ and CD8^+^ T cells. These findings were most pronounced in cirrhotic patients with systemic inflammation but fell short of reaching statistical significance when comparing against cirrhotic patients without systemic inflammation. In the former group TNF-α production in CD4^+^ and CD8^+^ T cells was reduced after stimulation with SEB, whereas there was no significant difference between the total cohort of cirrhotic patients and controls. After stimulation with the overlapping peptide pools for viral antigens EBNA1 and BZLF1, the number of responding T cells and the amount of TNF-α or IFN-γ production did not differ between the three pre-defined groups. However, cirrhotic patients with null-responses to EBV peptides had significantly higher serum IL-10 levels than responders to EBV peptides. Furthermore, TNF-α production in responding T cells was attenuated in patients with a high frequency of CD14^+^ HLA-DR^-^ monocytes.

**Conclusion:**

Our data suggest that bacterial translocation, endotoxaemia, inflammation and T cell activation in cirrhosis are accompanied by an increase in circulating anti-inflammatory cytokines, reduced monocytic MHC class II expression and attenuated cytokine production in T cells. These changes are likely to contribute to altered adaptive immune responses during infection or after vaccination.

## Background

Alterations of the immune system are very common in patients with end-stage liver disease and associated with an increased risk of infection and death [1-3]. Functional abnormalities of neutrophils and macrophages [4-8], natural killer cells [9], and the complement system [10] contribute to impaired innate immune responses and have been well described. There is also evidence to suggest that the adaptive immune response is defective in chronic liver disease, and cell-mediated immune responses after vaccination for hepatitis A, hepatitis B, or influenza are frequently attenuated in patients with decompensated liver cirrhosis. [11,12]. Although T cell responses towards hepatitis B virus and hepatitis C virus have been extensively studied [13,14], little is known about the T cell response to persistent other viruses such as Epstein-Barr Virus (EBV) as a marker of cellular immune function in cirrhosis.

In general, unresponsiveness of the adaptive immune system to antigens can be attributed to either a defect in antigen presentation by dendritic cells and macrophages, or to a suppressed or deficient T cell response. Cirrhosis predisposes to recurrent episodes of subclinical translocation of intestinal bacteria and bacterial products resulting in increased levels of endotoxin (lipopolysaccharide [LPS]) and tumour necrosis factor-alpha (TNF-α) [15]. Augmented by TNF-α, LPS is able to induce the secretion of interleukin-10 (IL-10) from Kupffer cells into the circulation [16,17], which can act as an inhibitor of cellular proliferation and T cell-mediated cytokine responses. This occurs in part through a down regulation of major histocompatibility complex (MHC) class II on monocytes/macrophages and inhibition of T-cell co-stimulatory pathways [18,19]. Indeed, reduced expression of MHC class II on monocytes has been observed in critically ill patients with cirrhosis [20,21] and in those with acute-on-chronic liver failure [22] and correlates with increased mortality. However, reduced monocyte HLA-DR expression also occurs in non-cirrhotic patients after trauma, during the systemic inflammatory response syndrome (SIRS) and sepsis [23,24]. SIRS is a frequent finding in patients with cirrhosis and is itself associated with poor outcome. [25] However, it is not known whether these phenotypic alterations occur in less severely ill cirrhotic patients (without SIRS) in association with endotoxaemia, and if present whether they contribute to functional T cell impairment.

To investigate the cellular immune status in cirrhosis, we studied T cell responses *in vitro* and their association with markers of bacterial translocation, serum IL-10, monocyte HLA-DR expression and T cell subsets in cirrhotic patients without SIRS. Healthy volunteers served as our controls.

## Methods

### Setting and participants

35 patients with liver cirrhosis presenting to our department between October 2009 and March 2010 and 14 self-declared healthy individuals were included. All subjects provided informed consent and local ethics committee approval was obtained. Liver cirrhosis was confirmed histologically, or through a combination of clinical, biochemical, and imaging data at the discretion of the investigator. Individuals were excluded if they met two or more criteria for systemic inflammatory response syndrome (SIRS) [26], if they were receiving immunosuppressive therapy, underwent surgical intervention within the last month, consumed alcohol within the last three days, experienced an episode of gastrointestinal bleeding, or underwent any endoscopic intervention within the last three days before inclusion. C-reactive protein (CRP) concentrations were measured as an indicator for systemic inflammation by routine laboratory analysis. Using the cut-off of 10 mg/l for cirrhotic patients defined by Papp *et al.*[27].

### Blood sampling and flow cytometry

9 ml heparinised whole blood and 9 ml EDTA whole blood samples (Sarstedt AG & Co, Nümbrecht, Germany) were collected, stored at 4°C, measured within 2 hours for cell surface receptors and stimulated with peptide pools within 3 hours. Briefly, 100 μl EDTA whole blood was stained for 20 min with fluorochrome-labelled monoclonal antibodies against HLA-DR (FITC, F7266; Dako, Hamburg, Germany), CD14 (PE, F0844; Dako), CD4 (FITC; F0766; Dako), CD8 (PE; R0806; Dako) and or CD25 (APC; 17–0259; eBioscience, Frankfurt, Germany) and washed in FACS buffer solution (PBS with 0.25% BSA and 0.02% sodium azide). When indicated, red blood cells were lysed using FACS lysing solution (BD Biosciences, Heidelberg, Germany) prior to staining. More than 50,000 PBMC were collected on a BD LSR II flow cytometer (BD Biosciences). Monocytes and lymphocyte populations were identified by the use of forward and right angle light scatter and by CD14, CD4 or CD8, respectively. The percentage of HLA-DR-expressing monocytes was calculated as the percentage of HLA-DR^+^ cells of total CD14^+^ cells; the percentage of CD25-expressing T cells was calculated as the percentage of CD25^+^ T cells of the CD4^+^ or CD8^+^ T cell population.

For intracellular cytokine staining, 500 μl heparinised whole blood was stimulated with peptide mixtures for 6 h at 37°C and 5% CO_2_ in the presence of 1 μg/ml co-stimulatory monoclonal antibodies to CD28 (L293) and CD49d (L25; BD Biosciences), and 10 μg/ml Brefeldin A (Sigma-Aldrich, Hamburg, Germany) as golgi-stop. The peptide pools (15-mers with 11 amino-acid overlaps) for Epstein-Barr nuclear antigen 1 (EBNA1) (130-093-613; Miltenyi Biotec, Bergisch Gladbach, Germany) or BamHI Z fragment leftward open reading frame number 1 (BZLF1) (130-093-611; Miltenyi) were added at a final concentration of 0.6 nM per reaction. The superantigen Staphylococcal enterotoxin B (SEB) was added at a final concentration of 1.5 μg/ml. After 6 hours incubation, 4 μl 0.5 M EDTA and 4.5 ml FACS lysing solution were added and cells resuspended in 1ml of permeabilising solution (0.25% BSA, 0.02% sodium azide, 0.5% saponin in PBS) for 10 min. After an additional centrifugation step, cells were stained with fluorochrome-labelled antibodies against CD4 (FITC) and CD8 (PE), IFN-γ (Pacific Blue; Invitrogen, Darmstadt, Germany), TNF-α (APC; Invitrogen) for 20 min at room temperature. Cells were washed and resuspended in 300 μl of FACS buffer solution (0.25% BSA and 0.02% sodium azide in PBS).

Negative samples without antigen stimulation were performed within each run. Positive cytokine responses were defined as a cell frequency of at least 2-fold above background (negative control without antigen) with a minimum of 50 cytokine-positive cells as previously described [28]. Cytometer setup and daily quality control of the instrument’s stability was performed by the cytometer setup and tracking module included into the FACS DiVa software package. Frequencies and median fluorescence intensities of labelled cells were calculated using FlowJo software (Tree Star, Ashland, OR, USA).

### Measurement of circulating interleukin-10 (IL-10) and lipopolysaccharide-binding protein (LBP)

Determination of serum concentrations of IL-10 and LBP was performed in 36 and 39 subjects, respectively. Serum LBP concentrations was measured in duplicate with the HK315 human LBP sandwich ELISA (Hycult Biotech, Uden, Netherlands; lower limit of detection 1 ng/ml) after dilution according to the manufacturer’s instructions. The standard curve was created from a 6-fold series of dilutions of a 50 μg/ml standard in duplicate. Serum IL-10 was determined using the ELISA MAX human IL-10 assay (Biolegend, San Diego, CA, USA; lower limit of detection 2 pg/ml) in duplicate according to manufacturer’s instructions. The standard curve was created from a 6-fold series of dilutions of a 250 pg/ml stock concentration. Measurements were performed using a photometric plate reader (VICTOR, Wallac, USA) at 460 nm.

### Statistical analysis

Baseline patient characteristics were reported as median and range for continuous variables, or as a frequency for discrete variables. Statistical analysis for nonparametric data was performed by using Mann–Whitney *U* test, Kruskal-Wallis test with post hoc Dunn’s test or Jonckheere-Terpstra test as appropriate. Bivariate correlation was either performed by the calculation of non-parametric Spearman’s correlation coefficient *R*_*s*_ or by Pearson’s linear correlation coefficient *R*. Statistical calculations were performed using SPSS v.16 (SPSS Inc., Chicago, IL, USA) and Graphpad Prism v. 5 (La Jolla, CA, USA).

## Results

### Patient characteristics

22 (63%) patients with cirrhosis had elevated CRP levels >10 mg/l. Causes were bacterial infections in 5 patients (2× SBP, 3× culture-positive urinary tract infections), inflammatory skin lesions in 3 patients, sterile pyuria in 4 patients and oedematous pancreatitis in 1 patient. An inflammatory focus could not be detected in 9 patients. None of the 13 cirrhotic patients with low CRP levels presented with clinically manifested bacterial infection.

**Figure 1:**
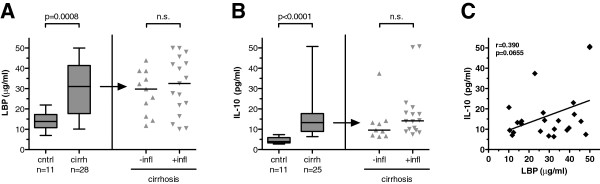
**Serum levels of lipopolysaccharide-binding protein (LBP) and interleukin-10 (IL-10) are increased in cirrhosis.** Box-plots of serum levels of (**A**) LBP and (**B**) IL-10 in healthy controls (cntrl) and cirrhotic patients (cirrh) are indicated (left). Scattered dot plots and medians are indicated for cirrhotic patients when stratified for inflammation (+/−infl) as evidenced by elevated CRP levels (right). *P* values in Mann–Whitney *U* test are indicated; n.s. = not significant. (**C**) Serum concentrations of IL-10 and LBP in cirrhotic patients tend to correlate in a linear fashion. Linear regression curve, Pearson product–moment correlation coefficient *R* and *P* value are indicated.

Cirrhotic patients that presented with signs of systemic inflammation more often had advanced liver disease indicated by ascites (Table 1). As expected, LBP serum levels were elevated in cirrhosis, but they did not differ with regards to the inflammation status as indicated by elevated CRP levels (Figure 1A). Concomitantly, IL-10 serum concentrations were elevated in patients with cirrhosis (Figure 1B) showing a trend of a positive linear correlation between serum IL-10 and LBP (Figure 1C).

**Table 1:** Baseline characteristics

**Characteristic**	**Controls (N=14)**	**Patients with cirrhosis without inflammation (N = 13)**	**Patients with cirrhosis and inflammation (N = 22)**	**P value**
**Years of age – median (range)**	39 (22–52)	55 (37–81)	65 (44–82)	<0.001
**Male sex – no. (%)**	8 (57%)	7 (54%)	14 (64%)	n.s.
**Positive Epstein-Barr virus serology – no. (%)**	12/14 (85%)	11/11 (100%)	25/25 (100%)	n.s.
**LBP serum concentration (μg/ml) – median (range)**	14 (7–22)	30 (12–44)	33 (10–50)	0.002
**IL-10 serum concentration (pg/ml) – median (range)**	4 (3–7)	10 (6–37)	14 (7–51)	<0.001
**Alcoholic aetiology of cirrhosis – no. (%)**		8 (62%)	15 (68%)	n.s.
**Child-Pugh stage A/B/C – no.**		6/4/3	2/3/17*	0.005
**Child-Pugh score – median (range)**		7 (5–13)	10 (6–13)*	0.005
**Ascites – no. (%)**		5 (38%)	19 (86%)*	0.007
**MELD score – median (range)**		12 (6–24)	16 (7–33)	n.s
**Laboratory values - median (range)**				
Total serum bilirubin - μmol/l		31 (5–504)	49 (7–324)	n.s
International normalized ratio		1.2 (0.9–2.0)	1.4 (1.1–2.2)*	0.029
Creatinine - μmol/l		76 (50–129)	91 (52–235)	n.s
Aspartate aminotransferase - μmol/l×s		0.78 (0.46–4.16)	1.23 (0.30–3.42)	n.s
Alanine aminotransferase - μmol/l×s		0.77 (0.36–2.68)	0.61 (0.24–1.10)	n.s
White blood cell count – Gpt/l		5.8 (2.4–7.2)	6.6 (3.6–16.8)*	0.034
C-reactive protein – mg/l		4.9 (2.0–10.0)	25.5 (12.6–91.9)*	<0.001
Serum Albumin – g/l		31 (19–40)	27 (17–35)	n.s.

P values in Kruskal-Wallis test or Mann–Whitney *U* test for continuous data or Fisher’s exact test for discrete data are indicated. * indicates a statistical significant difference between cirrhotic patients with and without elevated C-reactive protein levels.

MELD: Model for end-stage liver disease score, n.s.: not significant (P≥0.05).

### MHC class II expression on CD14^+^ monocytes is reduced in cirrhosis

**Figure 2:**
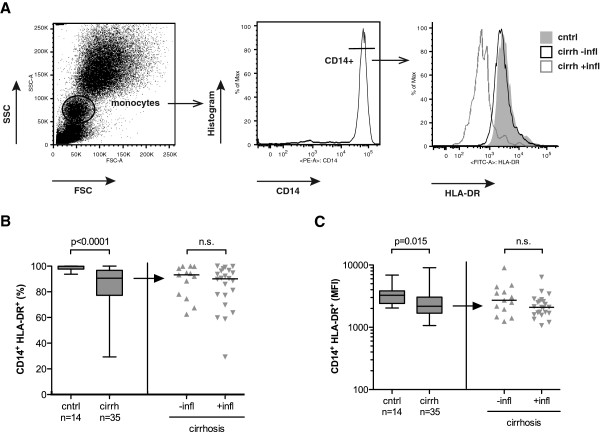
**Monocyte HLA-DR expression on CD14**^**+ **^**monocytes is reduced in cirrhosis.** Panel **A** displays the gating strategy to determine monocyte HLA-DR expression using flow cytometry. Monocytes were identified by forward and sideward scatter (left) and subsequent gating on CD14-positive cells (middle). The overlay histogram (right) demonstrates HLA-DR expression on CD14^+^ monocytes of three representative individuals from the control group (cntrl, grey area), from the cirrhosis group without inflammation (cirrh –infl, black line) and from the cirrhosis group with inflammation (cirrh +infl, grey line). Panels B and C display distribution and median of fraction of HLA-DR-expressing CD14^+^ monocytes (**B**) and of mean fluorescence intensity of HLA-DR^+^ CD14^+^ monocytes (**C**) from healthy controls and patients with cirrhosis (left) and from patients with cirrhosis when stratified for elevated CRP (right). *P* values in Mann–Whitney *U* test are indicated; n.s. = not significant.

The fraction of circulating monocytes within leukocytes was 6% (range: 3–12%) in controls, 8% (range: 3–20%) in cirrhotic patients without inflammation and 9% (range: 5–23%) in patients with cirrhosis and inflammation (*P*=0.008; Kruskal-Wallis test). In patients with cirrhosis, HLA-DR expression on CD14^+^ monocytes was reduced in cirrhosis (median 90.6%; range: 29.3%–100%) compared to controls (median 99.9%; range 93.7%–100%) (*P*<0.0001), whereas there was no significant difference with respect to the inflammation status in cirrhosis (Figures 2A and 2B). Accordingly, the surface HLA-DR expression on CD14^+^HLA-DR^+^ monocytes was markedly reduced in patients with cirrhosis as shown by a decreased mean fluorescence intensity (MFI; Figure 1C). However, there was no significant correlation between the fraction of HLA-DR^-^ monocytes or HLA-DR MFI on CD14^+^ monocytes with serum IL-10, serum LBP, Child-Pugh score or MELD score (*P*>0.4).

### The fraction of CD25^+^ T cells is increased in cirrhosis

**Figure 3:**
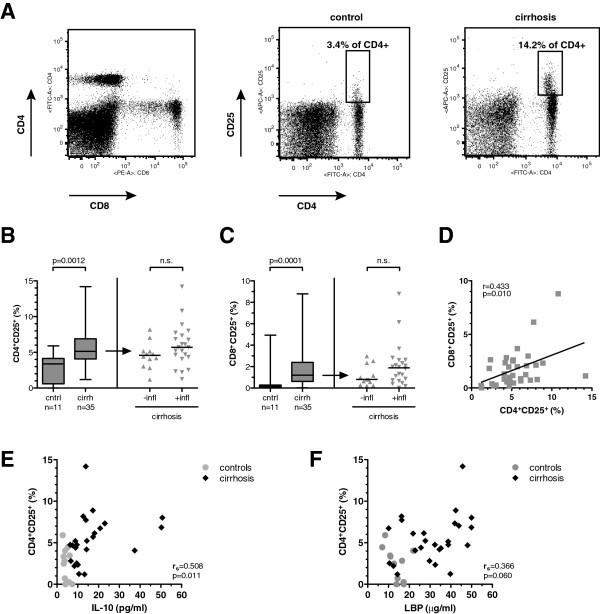
**Increased fraction of CD25**^**+ **^**T cells in cirrhosis.** (**A**) Gating strategy (left) and representative plots of CD25 expression on CD4 T cells in a healthy control (middle) and a patients with cirrhosis (right). Panels B and C display distribution and median of CD25-positive CD4^+^ T cells (**B**) and CD8^+^ T cells (**C**) in healthy controls and patients with cirrhosis (left) and in cirrhotics stratified for inflammation (right). *P* values in Mann–Whitney *U* test are indicated; n.s. = not significant. (**D**) Correlation of CD4^+^ CD25^+^ T cells and CD8^+^ CD25^+^ T cells in cirrhotic individuals. Linear regression curve, Pearson product–moment correlation coefficient *R* and *P* value are indicated. Panels E and F show the correlation of percentage of CD25-positive CD4^+^ T cells with IL-10 serum concentration (**E**) and LBP serum concentration (**F**) in patients with cirrhosis (black diamonds) and controls (grey circles), respectively. Spearman’s correlation coefficient *R*_s_ and *P* value are indicated for the cirrhotic patients.

Inversely to the monocyte fraction, circulating lymphocytes were lower in patients with cirrhosis and inflammation (median 26%; range 14%–79%) than in patients with cirrhosis without inflammation (38%; range 21%–95%) and in control subjects (51%; range 26%–66%) (*P*=0.014; Kruskal-Wallis test). Although the CD4/CD8 ratio did not differ between the three groups (*P*=0.439), patients with cirrhosis had a significantly increased fraction of CD25^+^ CD4^+^ T cells and CD25^+^ CD8^+^ T cells, a finding most pronounced in patients with evidence of inflammation (Figures 3A, 3B and 3C). The fractions of CD25-positive CD4^+^ and CD25^+^ CD8^+^ T cells correlated significantly in a linear fashion (*R*=0.433; *P*=0.01) (Figure 3D). Moreover, the fraction of CD25^+^ CD4^+^ cells positively correlated with serum IL-10 (*R*_s_=0.508; *P*=0.011) and tended to correlate with serum LBP (*R*_s_=0.366; *P=*0.060) (Figures 3E and 3F).

### The SEB-induced cytokine response is attenuated in cirrhotic patients with on-going inflammation

**Figure 4:**
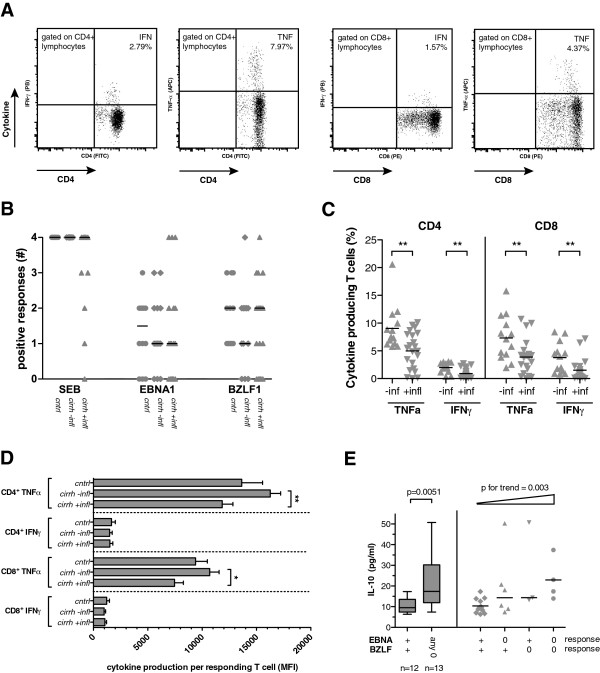
**T cell responses towards the viral antigens EBNA1 and BZLF1 and the superantigen SEB in cirrhosis.** (**A**) Representative plots of intracellular cytokine staining for TNF-α and IFN-γ in T cells after stimulation with SEB. (**B**) Distribution and median of T cell responses after stimulation with SEB, EBNA1 and BZLF1 in controls (circles), cirrhotic patients without inflammation (diamonds) and cirrhotic patients with inflammation (triangles). A positive response was defined as a cytokine-positive cell frequency of at least 2-fold above background (no antigen control) and the responses for TNF-α in CD4^+^ and CD8^+^ cells and for IFN-γ in CD4^+^ and CD8^+^ cells were added to a total score of 0 to 4. (**C**) Median and distribution of reacting CD4 T cells (left) and CD8 T cells (right) after SEB stimulation in cirrhotic patients with and without inflammation (+/−infl). ** indicates *P* values <0.01 in Mann–Whitney *U* test. (**D**) Mean florescence intensity (MFI) and standard error of mean are indicated for the intensity of TNF-α or IFN-γ response in CD4^+^ and CD8^+^ T cells after stimulation with SEB. P-values indicate results in Kruskal-Wallis test for each cytokine and T cell subset and asterisks indicate significance level in post-hoc Dunn’s multiple comparison test (* *P*<0.05; ** p<0.01). (**E**) Serum IL-10 levels from cirrhotic patients with a null response according to the aforementioned criteria (0) and from cirrhotic patients with at least one cytokine response (+) after stimulation with EBNA1 and BZLF1 are indicated (available from 25 cirrhotic patients). Patients were either stratified for the presence of a null response to EBNA1 or BZLF1 (left; *P* value in Mann–Whitney *U* test is indicated) and stratified for the combination of null-responses towards EBNA1 and BZLF1 (right; Jonckheere-Terpstra test demonstrates an increase in IL-10 in patients across the groups).

After stimulation of whole blood with the superantigen SEB, which cross-links the T cell receptor to MHC class II, all control subjects and all cirrhotic subjects without inflammation had a positive cytokine response for TNF-α and IFN-γ in both CD4^+^ and CD8^+^ T cell subsets (2-fold increase of responding cells), whereas 5 of 22 (23%) cirrhotic patients with inflammation had not (Figures 4A and 4B). The fraction of IFN-γ-producing or TNF-α-producing CD4^+^ and CD8^+^ cells after SEB differed significantly between cirrhotic patients with inflammation than in cirrhotic patients without inflammation (Figure 4C). Furthermore, TNF-α production in responding CD4^+^ and CD8^+^ T cells was lower in cirrhotics with inflammation than in cirrhotic patients without evidence of inflammation (Figure 4D). The number of TNF-α-producing CD4^+^ T cells and CD8^+^ T cells after SEB stimulation negatively correlated with CRP levels (*R*_s_=−0.439 [*P*=0.008] and *R*_s_=−0.443 [*P*=0.008], respectively) but not with IL-10 (*P*=0.709; *P*=0.408), MELD score (*P*=0.499; *P*=0.264) or Child-Pugh score (*P*=0.545; *P*=0.243) in non-parametric correlation, indicating cellular exhaustion in patients with severe acute phase reaction.

### Attenuated T cell responses to EBNA1 and BZLF1 are associated with higher interleukin-10 levels and low monocyte HLA-DR expression in cirrhotic patients

All cirrhotic patients and 12 of 14 control subjects were seropositive for EBV antibodies against EBNA as determined by immunoblot, indicating previous EBV infection. After stimulation of whole blood with overlapping peptide pools for the viral antigens EBNA1 and BZLF1, a broad spectrum of responses was observed in patients and control subjects. Figure 4B demonstrates the number of positive cytokine (TNF-α, IFN-γ) responses in CD4^+^ and CD8^+^ cells, represented as the sum of a positive response of each combination (score 0–4). The median number of positive responses after EBNA1 and BZLF1 exposition did not differ significantly between the three groups, which held also true in analyses performed separately for CD4 and CD8 T cell responses. Furthermore, intracellular cytokine production in responding T cells was not altered with respect to the three observation groups (data not shown). However, the fraction of patients with a null-response (score 0) towards EBNA1 or BZLF1 tended to be higher in patients with cirrhosis and inflammation (9/22 [41%] and 7/22 [32%], respectively) than in cirrhosis without inflammation (3/13 [23%] and 3/13 [23%]) or in controls (3/12 [25%] and 0/12 [0%]). Cirrhotic null-responders to any of the viral antigens EBNA1 or BZLF1 had higher IL-10 levels than cirrhotic patients with at least one positive response (*P*=0.005) (Figure 4E). IL-10 serum levels in cirrhosis significantly increased from responders through null-responders towards one of the viral antigens to null-responders to both viral antigens (*P* for trend = 0.003; Jonckheere-Terpstra test) (Figure 4E). Furthermore, patients with a low fraction of HLA-DR^+^ CD14^+^ monocytes <70% had a lower number of TNF-α-producing CD4^+^ T cells after stimulation with EBNA1 (median 0.02% vs. 0.05%; *P*=0.019). No correlation was found between low HLA-DR expression on CD14^+^ monocytes and TNF-α response in CD8^+^ T cells towards EBNA1 or BZLF1 peptides in cirrhotic patients (*P*=0.817 and *P*=0.976, respectively).

## Discussion

In this study we determined T cell responses towards EBV as a model to investigate the cellular immune function in patients with cirrhosis. Cirrhotic patients displayed elevated markers of bacterial translocation and increased T cell activation as well as reduced monocyte MHC class II expression and increased IL-10 serum levels. This phenotype was more pronounced in cirrhotic patients with systemic inflammation although did not reach statistical significance. We were not able to demonstrate significantly decreased antigen-specific T cell responses in the pre-defined patient cohorts. However, patients with diminished T cell responses towards the aforementioned viral proteins had significantly higher serum concentrations of IL-10 than patients with unaltered T cell responses.

It has been reported that HLA-DR expression on monocytes is increased on circulating classical and non-classical monocytes in patients with cirrhosis in the absence of bacterial infections and SIRS [29] but is reduced in critically ill cirrhotic patients [21]. In our cohort, patients with cirrhosis presented with reduced monocytic HLA-DR expression even in the absence of SIRS and sepsis. IL-10 impairs MHC class II recycling leading to decreased surface expression of HLA-DR [30], and a negative correlation of IL-10 and monocyte HLA-DR expression has been observed in patients with sepsis [31]. Indeed, we also observed high circulating IL-10 levels in cirrhotic patients which positively correlated with the degree of bacterial translocation as indicated by serum LBP concentration. Cells of monocyte-macrophage lineage secrete IL-10 in response to endotoxin [16] and their secretion capacity is not impaired but even pronounced in alcoholic cirrhosis [32]. The major source of circulating IL-10 in endotoxemia and abdominal infections are presumably Kupffer cells which are exposed to gut-derived LPS via the portal vein [16,17]. Besides elevated IL-10 concentrations, low serum levels of IFN-γ might have contributed to the impaired monocyte HLA-DR expression as observed in patients with alcoholic cirrhosis [33,34]. However, we did not observe a significant correlation of HLA-DR expression on CD14^+^ monocytes with liver function or stage of cirrhosis or circulating IL-10 levels.

In this study, we describe several characteristics in T cell phenotype and function in patients with cirrhosis. Despite a decrease in the lymphocyte population, the population of CD25-positive CD4^+^ and CD8^+^ T cells was expanded, and attenuated T cell responses to the SEB were more often observed in patients with cirrhosis and on-going inflammation. It remains unclear whether a reduction of MHC class II directly contributes to impaired antigen presentation to CD4 cells because even a low number of MHC class II molecules on antigen-presenting cells is sufficient to generate an effective T cell response [35] and a compensatory up-regulation of co-stimulatory molecules CD80 and CD86 has been observed on monocytes from cirrhotic patients [36]. Responses to SEB are dependent on HLA expression, since the superantigen cross-links MHC class II on monocytes with the T-cell receptor leading to a strong induction of TNF-α in T cells via protein kinase C [37]. Despite increased phenotypical markers of T cell activation in cirrhosis, we observed a reduced fraction of T cells with cytokine responses to SEB as well as reduced TNF-α production in CD4^+^ and CD8^+^ T cells after stimulation with SEB. The cytokine response toward the viral antigens EBNA1 and BZLF1 was not as distinct as the response to SEB with high inter-individual variability and no conclusive evidence for overall impaired T cell responses in cirrhosis. Besides down-regulation of MHC class II on monocytes, circulating IL-10 may also directly attenuate T cell responses by inhibition of the co-stimulatory CD28 signalling pathway [19]. Notably, we observed higher serum IL-10 concentrations in patients with a null response towards EBNA1 and/or BZLF despite the presence of anti-EBNA antibodies indicative of past EBV infection and immunoserological memory.

In addition to the observed pattern of attenuated T cell responses in patients with low monocytic HLA-DR expression or high circulating IL-10 levels, one can speculate that the presence of immune-regulatory subsets may also have contributed to attenuated T cell responses. Márquez *et al.*[36] reported that in patients with decompensated cirrhosis, an increase in CD25-positive effector CD4+ T cells was also accompanied by an increase of CD4^+^ CD25^high^ Foxp3^+^ regulatory T cells that may suppress T cell responses. This assumption, however, could not be corroborated in our study since we did not prove these cells to be CD127^low^ and Foxp3-positive. Moreover, a subset of CD14^+^ cells with low HLA-DR expression have recently been reclassified as myeloid-derived suppressor cells, and expand during infection or inflammation where they are capable of suppressing T cell responses and induce CD4^+^ CD25^+^ Foxp3^+^ regulatory T cells [38,39].

A further limitation of our study is that we did not correct for HLA restrictions as being responsible for non-response to stimulation with viral peptides. However, among the EBV antigens several epitopes of the latent protein EBNA1 and the immediate early lytic peptide BZLF1 are rather promiscuous in the MHC class I and II context [40]. Furthermore, our observations are in line with clinical observations: although cirrhosis may worsen the course of viral infections and vice versa [41,42], there is no strong evidence that cirrhosis itself predisposes to viral infections or other T cell defect-associated infections such as tuberculosis [43].

## Conclusions

Although this *ex vivo* study failed to provide evidence for a disturbed T cell response in cirrhotic patients in general, we did observe a pattern of attenuated responses towards viral antigens in patients that display low monocytic HLA-DR expression and/or increased serum IL-10 levels. Our data suggest that bacterial translocation, endotoxaemia, inflammation and T cell activation in cirrhosis are also accompanied by an increase in circulating anti-inflammatory cytokines, reduced monocytic MHC class II expression and attenuated cytokine production in T cells, which are likely to contribute to altered adaptive immune responses during infections or after vaccination *in vivo*.

## Competing interests

The authors who have taken part in this study declared that they do not have anything to disclose regarding conflict of interest with respect to this manuscript.

## Authors’ contributions

JP obtained the patients’ samples and performed the experiments. TB and JP analysed and interpreted the results, conducted literature search, performed statistical analysis and wrote the manuscript. AS conceived the study, supervised the work of co-authors, interpreted the results and revised the manuscript. OF participated in the design of the study and revised the manuscript critically for important intellectual content. All authors read and approved the final manuscript.

## Pre-publication history

The pre-publication history for this paper can be accessed here:

http://www.biomedcentral.com/1471-230X/13/37/prepub
